# Food insecurity and cognitive domains among older United States adults: Findings from the health and retirement study

**DOI:** 10.1002/alz.70480

**Published:** 2025-07-17

**Authors:** Elizabeth Ludwig‐Borycz, Heejin Lee, Lindsay H. Ryan, Steven G. Heeringa, Claire T. McEvoy, Julia A. Wolfson, Arun Agrawal, Kenneth M. Langa, Cindy W. Leung

**Affiliations:** ^1^ School for Environment and Sustainability University of Michigan Ann Arbor Michigan USA; ^2^ Department of Nutrition Harvard T.H. Chan School of Public Health Boston Massachusetts USA; ^3^ Institute for Social Research University of Michigan Ann Arbor Michigan USA; ^4^ Centre for Public Health, Institute of Clinical Sciences Queen's University Belfast Northern Ireland UK; ^5^ Department of International Health Johns Hopkins Bloomberg School of Public Health Baltimore Maryland USA; ^6^ Department of Health Policy and Management Johns Hopkins Bloomberg School of Public Health Baltimore Maryland USA; ^7^ Keough School of Global Affairs University of Notre Dame Notre Dame Indiana USA; ^8^ Department of Internal Medicine University of Michigan Medical School, Taubman Health Care Center Ann Arbor Michigan USA

**Keywords:** Alzheimer's and dementia, cognitive domains, cognitive function, executive function, food Insecurity, health and retirement study

## Abstract

**INTRODUCTION:**

The number of cases of Alzheimer's disease and related dementias (ADRD) is expected to triple in the United States in the next three decades. Food insecurity may be a risk factor for cognitive decline. However, studies exploring the relationship between food insecurity and individual cognitive domains within the United States remain limited.

**METHODS:**

Data came from 1410 participants from the nationally representative Health and Retirement Study. Food insecurity was assessed in 2013, and cognition was assessed using the Harmonized Cognitive Assessment Protocol in 2016.

**RESULTS:**

The prevalence of food insecurity was 13.1%. After adjusting for all covariates, food insecurity was inversely associated with executive functioning (β = −1.47, 95% CI −2.65, −0.28) but not significantly associated with memory, language, visuospatial functioning, or orientation.

**DISCUSSION:**

Further research is needed to understand how food insecurity may influence executive function over time and to explore potential underlying mechanisms for this association.

**Highlights:**

- After adjusting for age and sex with Bonferroni correction, food insecurity was inversely associated with scores on three of the five cognitive domains: memory (β = −2.67 95% CI −4.41, −0.94); executive functioning (β = −3.50 95% CI −4.62, −2.37); visuospatial (β = −3.18 95% CI −6.13, −0.24.
- After additional adjustment for other covariates, the inverse association between food insecurity and executive functioning remained statistically significant (β = −1.47 95% CI −2.65, −0.28).
- Other associations were attenuated and lost statistical significance.

## 1 BACKGROUND

The number of Americans with Alzheimer's disease and related dementias (ADRD) is expected to triple in the next three decades to 14 million.^1^ ADRD is a leading cause of disability and premature mortality in the United States.^2^ As ADRD progresses, cognitive function worsens along with impairments in daily activities and the development of neuropsychological symptoms.^3^ ADRD is more prevalent among women, Hispanic, and Black populations.^4^, ^5^

Food insecurity (FI) is limited access to nutritious food due to insufficient economic or other resources.^6^ FI is more prevalent among lower‐income adults, women, and individuals of minority racial and ethnic backgrounds.^6^ FI is associated with ADRD risk factors, such as poor diet,^7^, ^8^ cardiometabolic health,^9^, ^10^, ^11^ and mental health.^12^, ^13^ Additionally, FI itself may be a risk factor for ADRD. Studies in the National Health and Nutrition Examination Survey (NHANES)^14^, ^15^ and cross‐sectional studies from several countries found that FI is associated with lower cognitive function in older adults,^16^, ^17^ yet fewer longitudinal studies have examined its associations. Most studies conducted were relatively small and specific populations, such as Puerto Ricans in Boston^18^, ^19^ and human immunodeficiency virus (HIV) patients in Miami,^20^ limiting the generalizability to the broader US population. Recently, longitudinal studies explored the relationship between FI and cognitive function using national data. Research using the Health and Retirement Study (HRS) found that FI was associated with lower levels of cognitive functioning, increased likelihood of cognitive impairment, increased dementia risk, poorer memory function, and faster memory decline among older US adults.^21^, ^22^, ^23^, ^24^, ^25^ Another HRS study found that among participants eligible for Supplemental Nutrition Assistance Program (SNAP, food provision), those who used it had a protective effect on memory decline compared to those who did not.^26^ A separate US study found food sufficiency and SNAP participation were protective of cognitive decline.^23^ Despite these global associations, nationally representative studies exploring the relationship between FI and multiple individual cognitive domains, such as memory, executive functioning, language, visuospatial functioning, and orientation, remain rare for the United States.

A nuanced examination of the five cognitive domains: memory, executive functioning, language, visuospatial functioning, and orientation will provide a better opportunity to locate the mechanisms through which FI drives ADRD by isolating the cognitive domains affected. There are a variety of possible mechanisms that could drive a negative association between FI and cognitive domains, poor nutrition, stress, physical activity, anxiety, and/or depression.^27^ People who experience FI have poorer overall diet quality and could be low in B12, B6, folate, thiamine, or vitamin D, which contributes to healthy memory and language, and low in B12, iron, vitamin D, or omega‐3 fatty acids, which contribute to healthy executive function, visuospatial functioning, and orientation.^28^, ^29^, ^30^, ^31^, ^32^ A 2021 review found that, FI was negatively associated with executive function, verbal memory, and visuospatial abilities; however, most of these studies were cross‐sectional.^33^ Since then, a 2023 cross‐sectional study was conducted in India, which found no association between FI and memory, but did observe negative associations with orientation, executive function, object naming, and arithmetic function.^34^ This study should be replicated in the United States to help determine if findings are consistent across populations and identify driving mechanisms that can be targeted for intervention.

Since FI, ADRD, and mild cognitive impairment (MCI) are more common among women and people of color, it is important to consider gender and race/ethnicity as effect modifiers between food security and cognitive domains. Women bear a larger social burden of food preparation^35^ and will scale back on their food consumption to prioritize others.^36^ People of racial/ethnic minorities already deal with stigma and discrimination, and the combined level of stress and stigma due to FI and racism could further exacerbate outcomes.

We aim to evaluate the associations between household FI and multiple cognitive domains using a nationally representative sample of older adults in the United States to address some of the prevailing gaps in the literature. We hypothesize that FI is associated with lower performance in all five cognitive domains, and that this association is exacerbated for people of color and women.

## 2 METHODS

### 2.1 Study population

The HRS is a nationally representative longitudinal study of adults aged 51 or older in the United States and has a sample size of more than 20,000 (NIA U01AG009740). Data are available at http://hrsonline.isr.umich.edu and have been described in detail in Sonnega et al., 2014.^37^ HRS began in 1992 recruiting cohorts of adults over the age of 50. It continues to recruit new cohorts every 6 years.^37^ Every 2 years, an HRS core survey is administered via in‐person, telephone, or Web collecting information on participants’ income and wealth, health and use of healthcare services, work and retirement, family connections, and cognitive function.^37^ Socio‐demographic and health variables for this analysis were drawn from the HRS 2012 core dataset and linked to the Cross‐Wave Tracker File for variables on complex sampling design and combined with the RAND 1992‐2014 Cross‐Wave Income and Wealth data set, along with two supplemental studies: 2013 Health Care and Nutrition Study (HCNS) and the 2016 Harmonized Cognitive Assessment Protocol (HCAP) Project.^37^, ^38^ The flow chart of the sample is shown in Figure S1. HRS was conducted in accordance with the Declaration of Helsinki guidelines and the University of Michigan Health Sciences/Behavioral Sciences Institutional Review Board (IRB).

### 2.2 Household food insecurity

The HCNS was a mail survey administered between November 2013 and May 2014 to a subsample of HRS participants (*n* = 8073). The survey collected food and nutrition data, including information about FI. Household FI was assessed using methods published by Aljahdali et al., 2024.^39^ In brief, food security was assessed using the Six‐Item Short Form of the Food Security Survey Module.^40^ Food security was determined using scores of 1 to affirmative responses and 0 to negative responses for each of the six items assessing perceptions and behaviors related to insufficient resources to purchase or acquire foods. The six items were summed to create a score ranging from 0 to 6. The score was then categorized according to United States Department of Agriculture (USDA) guidelines: food security 0–1 affirmative responses and FI ≥2 affirmative responses.^40^

### 2.3 Cognitive domains

Our study used HRS cognition data collected in 2016 in the Harmonized Cognitive Assessment Protocol (HCAP).^37^ The HCAP cognitive data have more breadth and depth than those collected in the HRS Core. HCAP interviews included the Mini‐Mental State Examination (MMSE), HRS Telephone Interview for Cognitive Status (HRS‐TICS), Consortium to Establish a Registry for Alzheimer's Disease (CERAD) word list learning and recall (immediate and delayed), retrieval fluency, letter cancellation, backward count, 10/66, story memory (immediate and delayed), CERAD word list recognition, CERAD constructional praxis (immediate and delayed), Symbol‐Digit Modalities Test (SDMT), Wechsler Memory Scale, Fourth Edition (WMS IV) logical memory recognition, HRS number series, Raven's standard progressive matrices, Trail Making Test (A and B), Center for Epidemiological Studies Depression Scale (CES‐D depressive symptoms), and smell test (National Social Life, Health, and Aging Project [NSHAP]). The HCAP in 2016 administered about 1 hour of neuropsychological testing to an HRS sub‐sample of adults aged 65 or older (*n* = 3496) with multiple measures across five cognitive domains: memory, executive function, language, visuospatial functioning, and orientation (see Table S1). Cognitive domains were defined following factor analysis model, factor scoring and T‐score derivations developed by Jones et al., 2024^41^ for the HCAP and Lin et al., 2023 (Preprint, not yet peer‐reviewed). Measures to assess memory included the 10‐word recall (delayed), 3‐word recall (delayed), Logical memory II (delayed), Story recall—brave man (delayed), 10‐word recall (recognition), Logical memory (recognition), and Constructional praxis (delayed). Measures to assess executive functioning included Standard Progressive Matrices, Number series, Trial Making Test part B, SDMT, Trial Making Test part A, Backwards spelling, Backwards counting, and Letter cancellation. Measures to assess language included animal naming, Object naming TICS, Object naming MMSE, 0–2, write a sentence, read and follow command, Object naming CSI‐D. Measures to assess the visuospatial cognitive domain included the constructional praxis (immediate) task, adjusted to a T‐score metric. Measures to assess orientation included orientation to time and place. In accordance with Manly et al., 2022^43^ we standardized the sum of all tests in each of the five cognitive domains to a mean of 50 and a standard deviation of 10.

**RESEARCH IN CONTEXT**

1. **Systematic review**: The number of cases of Alzheimer's disease and related dementias (ADRD) is expected to triple in the United States in the next three decades. Food insecurity may be a risk factor for cognitive decline. Studies exploring the relationship between food insecurity and individual cognitive domains (CD), such as memory, executive functioning, language, visuospatial function, and orientation remain rare in the United States.
2. **Interpretation**: The value in our study lies in that it is the first nationally representative study of older US adults to assess the associations between household food insecurity and multiple cognitive domains. Results showed that food insecurity was significantly associated with worse executive functioning; most associations with other cognitive domains were not significant.
3. **Future directions**: Further research is needed to understand how food insecurity may influence executive function over time and to explore potential underlying mechanisms for this association.

### 2.4 Covariates

Covariates were chosen for their potential to confound the association between FI and cognition and based on prior literature.^21^, ^25^ Self‐reported sociodemographic characteristics included: age, sex, race, and ethnicity (White, Black, Hispanic, or other–including American Indian Alaskan Native, Asian, Native Hawaiian, Pacific Islander, and other racial/ethnic groups), years of education (less than high school, high school graduate or equivalent, some college or college graduate, or post‐college), total net worth of all assets (excluding non‐primary residences),^38^ marital status (married, separated/divorced/widowed, never married), and childhood socioeconomic status (SES; a validated measure that averaged childhood financial, human, and social capital).^44^ Health status included: cardiovascular disease, depression, body mass index (BMI; underweight, normal, overweight, and obese), and the Langa–Weir Classification of Cognitive Function for assessing dementia status at baseline (Normal, Cognitively Impaired but not Demented, and Demented). Health behaviors included: moderate or vigorous physical activity (hardly ever or never, a few times per month, and once a week or more), smoking status, and alcohol consumption in grams per day. All covariate data were drawn from the 2012 core HRS or 2013 HCNS surveys.

### 2.5 Statistical analysis

All statistical analyses accounted for the HRS's complex study design. Since this study is the intersection of two HRS sub‐samples, neither of the HRS‐provided weights are guaranteed to map our sample back to the HRS core population. As such, the 2016 HCAP weights needed to be adjusted to account for the exclusions (HCAP cases with no matched HCNS interview). Therefore, we used propensity score‐based weighting, treating 2016 HCAP as the reference sample to avoid post‐2013 mortality concerns. Propensity‐based weights were created by logistic regression to model the probability that an HCAP interview also had a matched HCNS interview. To avoid extreme weights from cases at the far lower tail of the distribution, we assigned the propensity values for all cases to 10 deciles. We created the propensity adjustment weight for each sample case by taking the reciprocal of the mean propensity for the decile in which the cases' modeled propensity lay. We then created the population‐adjusted weight by multiplying the 2016 HCAP weight for each of the cases by its propensity weight.

Descriptive statistics were presented as means and standard error (SE) or percentages for continuous and categorical sociodemographic variables, respectively, stratified by food security status. Independent sample *t*‐tests and chi‐squared tests were conducted to examine the differences in the stratified descriptive analyses. Means and SE were presented for each cognitive domain separately by the food security status. To examine the associations between food security status and each cognitive domain (memory, executive function, language, visuospatial, and orientation), we used multivariate linear regression models with Bonferroni corrections to reduce the risk of type I error, which instead provides a conservative estimate when testing multiple hypotheses. The first model adjusted for age and sex; the second model additionally adjusted for race/ethnicity, total net worth, education, and marital status; and the third model adjusted further for physical activity, BMI, smoking status, cardiovascular disease (CVD), alcohol consumption, and depression status. We also tested for effect modification by sex and race/ethnicity, first by testing the interaction term and then conducting stratified analyses for (1) FI and sex and (2) FI and race/ethnicity on all five cognitive domains. Furthermore, we performed sensitivity analyses to test the robustness of our findings by (1) including marginal food security in the food insecure category rather than food secure, and (2) adjusting for childhood SES on top of all covariates in model three. We used SAS 9.4 (SAS Institute, Cary, NC) for data analysis.

## 3 RESULTS

The analytic sample comprised 1410 study participants, 13.1% (*n* = 185) of whom were food insecure and 86.9% (*n* = 1225) were food secure (Table 1). In 2013, the average age of study participants was 69.4 years (SE = 0.3), 54.4% were female, and 83.4% identified as non‐Hispanic White. Adults with FI were on average younger (mean 67.9 years, SE = 0.6) than adults with food security (mean 69.6 years, SE = 0.3) (*p*‐value = 0.014). Food‐insecure participants were more likely to be Black (14.6%), Hispanic (19.8%), or another minority racial or ethnic group (3.3%) than their food secure counterparts (Black 13.16%, Hispanic 19.8%, or another minority racial or ethnic group 3.4% [*p*‐value < 0.0001]). Educational attainment was lower for FI participants with the minority having some college education or greater (34.1%) than for food‐secure participants with the majority (57.1%) having some college education or greater (*p*‐value < 0.0001). The average net worth of FI participants ($59,966 [SE = $10,697])was 13.8 times lower than that of food secure participants ($826,798 [SE = $173,362]) (*p*‐value < 0.0001). Food‐insecure participants in 2013 were less likely to be married (49.3%) than those who were food‐secure (71.9%) adults (*p*‐value = 0.0010). Alcohol consumption was lower for FI adults ([2.1 g (SE = 0.54]) compared to food‐secure adults [7.88 g (SE = 0.61)] (*p*‐value < 0.0001). More food‐secure adults (76.3%) engaged in regular moderate or vigorous physical activity once a week or more, compared to their food‐insecure counterparts (59.3%) (*p*‐value < 0.0068). Adults experiencing FI were more likely to be obese (58.4% *p*‐value = 0.0031), a smoker (20.3% *p*‐value < 0.0001), have CVD (45.6% *p*‐value = 0.0001), and depression (20.3% *p*‐value < 0.0001) than their food secure counterpart (43.7%, 7.2%, 26.7%, and 4.6%, respectively).

**TABLE 1:** Sample characteristics in the HRS population.

	Mean (SE) or %		
Characteristic^a^	Total (*n* = 1410)	Food Secure % (*n* = 1225)	Food Insecure % (*n* = 185)	*p*‐Value
Age (years)	69.44 (0.28)	69.63 (0.31)	67.91 (0.57)	0.0139
Gender				0.2831
Female	54.41	55.05	49.15	
Race				<0.0001
White	83.43	85.86	63.68	
Black	7.15	6.41	13.16	
Hispanic	7.67	6.18	19.75	
Other^b^	1.75	1.55	3.41	
Years of education				<0.0001
Less than high school	11.68	9.50	29.48	
High school graduate	33.71	33.38	36.40	
Some college/college graduate	36.85	38.78	21.1712	
Post‐college	17.7565	18.3472	12.94	
Total net worth	742963 (155774)	826798 (173362)	59966 (10697)	<0.0001
Marital status				0.0015
Married	69.44	71.91	49.34	
Separated/divorced/widowed	25.31	23.56	39.52	
Never married	5.25	4.53	11.13	
Alcohol consumption (grams)	7.25 (0.57)	7.88 (0.61)	2.10 (0.54)	<0.0001
Physical activity (moderate or vigorous)				0.0068
Hardly ever or never	15.4299	13.8669	28.1631	
A few times per month	10.11	9.80	12.59	
Once a week or more	74.46	76.33	59.25	
BMI				0.0031
Underweight	0.67	0.73	0.22	
Normal	18.24	19.48	8.17	
Overweight	35.82	36.13	33.25	
Obese	45.27	43.66	58.36	
Smoking status				<0.0001
Smoker	8.58	7.14	20.32	
Cardiovascular disease status				0.0001
Cardiovascular disease	28.78	26.72	45.56	
Depression status				<0.0001
Depression	6.27	4.55	20.33	

Abbreviations: BMI, body mass index; HCNS, 2013 Health Care and Nutrition Study; HRS, Health and Retirement Study; SE, standard error.

^a^ All characteristics were drawn from the 2012 core HRS or 2013 HCNS surveys.

^b^ Other includes American Indian, Alaskan Native, Asian, Native Hawaiian, Pacific Islander, and other racial/ethnic groups.

The associations between FI and the five standardized (mean of 50 and SD of 10) cognitive domains are presented in Table 2, where memory, executive function, and visuospatial were statistically different. For memory, the mean score for food‐insecure participants was 49.28 (SE = 0.62) and for food‐secure participants was 51.44 (SE = 0.22). For executive function, the mean score for food‐insecure participants was 48.01 (SE = 0.4) and for food‐secure participants was 51.13 (SE = 0.14). For visuospatial, the mean score for food‐insecure participants was 49.10 (SE = 1.12) and for food‐secure participants was 51.82 (SE = 0.33). After adjusting for age and sex (model 1), FI was inversely associated with memory, executive function, and visuospatial cognitive domains: memory (β = −2.67, 95% CI −4.41, −0.94); executive functioning (β = −3.50, 95% CI −4.62, −2.37); and visuospatial (β = −3.18, 95% CI −6.13, −0.24). After additional adjustment (model 2) for other sociodemographic covariates (race/ethnicity, total net worth, education, and marital status), the inverse association between FI and executive functioning remained statistically significant (β = −1.87, 95% CI −3.16, −0.58); other associations were attenuated and lost statistical significance. After additional adjustments (m0odel 3) for physical activity, BMI, smoking status, CVD, alcohol consumption, and depression status, the inverse association between FI and executive functioning was attenuated slightly but remained statistically significant (β = −1.47, 95% CI −2.65, −0.28).

**TABLE 2:** Associations between food insecurity and cognitive domains (*n* = 1410).

			β (95% CI)		
Parameter	Food secure Mean (SE)	Food insecure Mean (SE)	Model 1^**^, ^a^	Model 2^**^, ^b^	Model 3^**^, ^c^
Memory^*^	51.44 (0.22)	49.28 (0.62)^d^	−2.67 (−4.41, −0.94)	−0.77 (−2.59, 1.07)	−0.49 (−2.23, 1.25)
Executive Functioning^*^	51.13 (0.14)	48.01 (0.40)^d^	−3.50 (−4.62, −2.37)	−1.87 (−3.16, −0.58)	−1.47 (−2.65, −0.28)
Language^*^	50.46 (0.15)	49.22 (0.59)	−1.40 (−3.09, 0.29)	−0.20 (−1.71, 1.31)	0.16 (−1.27, 1.60)
Visuospatial^*^	51.82 (0.33)	49.10 (1.12)^d^	−3.18 (−6.13, −0.24)	−0.50 (−3.38, 2.37)	0.23 (−2.42, 2.89)
Orientation^*^	50.80 (0.26)	50.80 (0.26)	−1.83 (−3.73, 0.08)	−0.67 (−2.54, 1.21)	−0.49 (−2.68, 1.71)

Abbreviations: BMI, body mass index; CI, confidence interval; CVD, cardiovascular disease; HCNS, 2013 Health Care and Nutrition Study; HRS, Health and Retirement Study; SE, standard error.

* Standardized to mean 50 and SD of 10.

** All covariates were drawn from the 2012 core HRS or 2013 HCNS surveys.

^a^ Adjusted for age and sex with Bonferroni correction.

^b^ Adjusted for age, sex, race/ethnicity, total net worth, education, and marital status with Bonferroni correction.

^c^ Adjusted for age, sex, race/ethnicity, total net worth, education, marital status, physical activity, BMI, smoking status, CVD, alcohol consumption, and depression status with Bonferroni correction.

^d^ Means significantly different at *p* < 0.05.

In the fully adjusted model (model 3), there was a significant effect modification by sex for the association between FI and memory (P‐interaction = 0.0366) (Table S2). In stratified analyses, there was an inverse association between FI and memory for males (β = −1.79, 95% CI −4.35, −0.77); there was no significant association for females (β = 0.76, 95% CI −1.30, 2.82). Similarly, there was significant effect modification by sex for the association between FI and executive function (P‐interaction = 0.0464). In stratified analyses, the magnitude of the association between FI and memory was greater for males (β = −2.14, 95% CI −3.95, −0.33) than for females (β = −1.05, 95% CI −2.21, −0.11). For the remainder of the associations between FI and cognitive domains, the differences by sex were null. There was also no evidence of effect modification by race and ethnicity for any of the associations between FI and cognitive domains (Table S3).

In sensitivity analyses, results changed marginally. The first sensitivity analysis included marginal food security in the food insecure category rather than food secure (Table S4), which maintained the negative association between FI and executive function in models 1 and 2 but lost statistical significance in the fully adjusted model 3. The second sensitivity analysis presented in Table S5 further adjusted for childhood SES in the fully adjusted model and found nearly identical results to the main analysis (β = −1.58, 95% CI −2.98, −0.18), with the association between FI and executive functioning remaining statistically significant.

## 4 DISCUSSION

The objective of this study was to examine the associations between household FI and individual cognitive domains using a nationally representative sample of US older adults. We hypothesized that FI would be associated with lower cognitive performance on multiple cognitive domains. Our results showed that FI was significantly associated with worse executive functioning. Our sensitivity analysis suggests that the association is mostly driven by the more severe levels of FI. We also note that the HRS participants with FI were, on average, younger than those with food security. Despite adjusting for age in the analyses, the FI and executive function association persisted, suggesting that the difference in executive function by FI groups is worth further exploration. Putting this into perspective with other risk/protective factors, we found that the negative effects of FI on executive function were comparable to around 8 additional years of age in our sample or about the same level of magnitude that protects someone with more than 4 years of college compared to someone with only a high school diploma (Table S6). Our second hypothesis that FI may have a greater negative impact on cognitive domains for people of color and women proved false. Sex was an effect modifier of the relationship between FI and memory and executive function, but, in contrast with our hypothesis, it had a greater negative impact on men. Race and ethnicity were not modifiers.

Other studies have found that FI is negatively associated with overall cognitive function.^14^, ^15^, ^18^, ^19^, ^20^, ^21^ Our finding that FI is associated with worse executive function corroborates prior studies. A Boston cross‐sectional study of 1358 Puerto Rican adults found an inverse association between FI and executive function, with no association between memory or attention factors.^18^ In the longitudinal follow‐up to this study, FI was associated with a faster decline in executive function but not memory.^19^ Our results showed a similar prospective association with both executive function and memory. In the 2011–2014 NHANES, FI was inversely associated with cognitive tasks that assessed processing speed, sustained attention, verbal fluency, working memory, and immediate learning ability—all processes related to executive function.^15^ The present analysis builds on these findings with a nationally representative sample rather than a regional sample (Boston) and with lengthier and more precise measures of executive function than in NHANES. Our findings continue to highlight the need for more longitudinal research on FI and executive function to better understand the nature of this association.

Potential mechanisms that link FI to executive function are increased stress and allostatic load, lower fruit and vegetable consumption, poorer nutrition, reduced physical activity, depression, and anxiety.^27^ Exposure to FI has been shown to increase stress hormones, which may undermine the ability of the prefrontal cortex to perform cognitive processes such as executive function.^45^, ^46^, ^47^, ^48^, ^49^, ^50^, ^51^, ^52^, ^53^ FI is also associated with poorer nutrition, including lower fruit and vegetable consumption. Greater fruit and vegetable consumption and higher diet quality have been associated with slower rates of cognitive decline.^54^, ^55^, ^56^ This could be due to the higher antioxidant content in fruits and vegetables, which can prevent cognitive decline.^55^, ^56^ The high folate content in vegetables could also be a reason for this relationship as low levels of folate can lead to increased homocysteine, which has a neurotoxic effect and is associated with increased dementia and cognitive decline.^56^ Physical activity, particularly aerobic exercise, is another health behavior associated with improved cognitive function through less age‐related gray and white matter loss and with fewer neurotoxic factors, including beta‐amyloid and excessive glucose.^57^ Physical activity reduces the likelihood of vascular diseases, improves respiratory function, stimulates growth factors, particularly brain‐derived neurotrophic factor and insulin‐like growth factor‐1, and downregulates oxidative stress and inflammatory responses.^57^ FI is also associated with reduced physical activity, providing another possible mechanism between FI and reduced executive functioning.^57^, ^58^ Previous research has shown that the associations between FI and cognitive decline are partially mediated through depression and anxiety, both of which may be additional mechanisms driving the decline in executive function.^59^, ^60^, ^61^ Anxiety and depression may impact executive function through increased inflammation or changes in gray matter.^62^, ^63^

We hypothesized that FI would be negatively associated with all five cognitive domains, however, the only significant association was with executive function. Other research has assessed the relationship between food security and memory but largely found a null association, similar to our results.^27^ Stress may not affect memory as much as expected.^64^ Although it has been suggested that FI may reduce language, visuospatial, and orientation cognitive domains, little research has been conducted that supports this relationship in observational or clinical studies.^14^, ^17^ Considering our study is one of the first that assessed each of five cognitive domains, our findings suggest that the relationship between FI and cognitive function may primarily be driven by executive function rather than the other cognitive domains.

The growing body of research linking FI to cognitive impairment among older adults suggests that efforts to reduce FI among older adults could potentially lead to cognitive benefits. SNAP is the largest nutrition assistance program and works by providing benefits to purchase food to low‐income Americans. Households with older adults have lower participation rates in SNAP despite meeting eligibility criteria.^65^ Community efforts to increase SNAP enrollment among older adults could have benefits beyond improved food security. In addition to food assistance, the Federal and State governments can also work to improve food security through more general need‐based assistance. For example, indirectly focusing on freeing up a proportion of people's fixed monthly income to utilize on food by creating more affordable housing options for seniors, such as the Low Income Housing Tax Credit (LIHTC) program, to help with the cost of senior living.^66^ LIHTC lowers the cost of housing such that it reduces the risk that adults have to choose between paying rent and buying food, thereby improving food security for seniors. In addition to federal and local programs, community support is also important. For seniors, challenges like limited mobility, cognitive decline, or difficulties accessing information might make it harder for them, social workers or community health workers could help guide and connect them to appropriate resources.^67^

The effect modification analysis found that men were more sensitive to the adverse effects of food insecurity on memory and executive function. Men may experience greater psychological distress as a result of food insecurity than women due to gender norms and social and cultural expectations to financially support the household.^68^ This, in conjunction with the social stigma attached to help‐seeking behavior for men, may lead men to underreport psychological distress and nutritional needs, thereby exacerbating their experiences of food insecurity rather than finding relief through reaching out to seek community‐based support.^69^ More research into the gender differences of food security and memory and executive function should be conducted to examine and better understand this finding in other populations.

A major strength of this analysis is the large sample size and the generalizability of the findings to US older adults. Additionally, the six‐item food security measure used has a high specificity and sensitivity with minimal bias.^40^ Furthermore, the HCAP cognitive domain measures have greater breadth and depth than those in HRS Core data and were selected with a high degree of compatibility in mind to allow for comparisons between prior HRS studies and its international partner studies in Mexico, India, England, Ireland, Chile, China, and South Africa.^37^ A primary limitation of this study is the single administration of a validated food security measurement tool in 2013 and the administration of the HCAP in 2016. This limitation is tempered by the measurement of FI in 2013 before the administration of the HCAP in 2016, thus establishing temporality in the association and reducing the possibility of recall bias. However, FI can be a dynamic condition, and transitioning to or from food security could impact mechanisms between FI and cognitive domains that lead to differences in cognitive change. A recent study found that more older adults in the United States are experiencing recurrent or chronic FI which may have led to an underestimation of the association in the present analysis.^70^ Further studies with repeated validated food security measures may be needed to examine these associations. Since both HCNS and HCAP have selective criteria for participation, the intersection of participants in both studies is significantly smaller than the full HRS sample and can be selective. As such, the estimated associations between FI and cognitive domains might be biased. To minimize this bias and improve the estimate, we used propensity score‐based weighting, treating 2016 HCAP as the reference sample, yet it is possible that our findings may not fully represent populations that were under‐sampled or those with systematic differences that were not captured in the propensity score model. Additionally, the smaller sample size in this analysis, particularly within specific subgroups, may have been underpowered to detect significant subgroup differences by sex and race/ethnicity, and larger studies with these rigorous measures are needed for further investigation. Finally, we cannot rule out residual confounding factors.

We found that FI was associated with worse executive function. Further research is needed to understand how FI may influence executive function over time and to explore underlying mechanisms. This growing body of research is critically important to inform programs and policies that alleviate FI and that may improve cognition.

## CONFLICT OF INTEREST STATEMENT

No co‐author has any outside interests to disclose. Author disclosures are available in the supporting information.

## CONSENT STATEMENT

All human subjects provided informed consent.

## Supporting information



Supporting Information

Supporting Information
