# Supplementary material for: Food insecurity and cognitive domains among older United States adults: Findings from the health and retirement study
Source: Alzheimers Dement. 2025 Jul 17;21(7):e70480. doi: 10.1002/alz.70480 (PMC12268310; doi:10.1002/alz.70480)
Supplement: Supplementary file 1 — Supporting Information [file ALZ-21-e70480-s001.docx]

**Supplemental Figure 1: Flow chart of the sample**

2016 Harmonized Cognitive Assessment Protocol (HCAP)

**n= 3,496**

2013 Health Care and Nutrition Study (HCNS)

**n=8,073**

Participants who completed both the 2013 HCNS and the 2016 HCAP with non-missing food security status **n=1,506**

Participants with possible dementia (n=44) at baseline were excluded **n=1,462**

Participants with missing values for covariates (n=23) [physical activity (n=9), smoker (n=8), CVD (n=8), marital status (n=8), total net worth (n=8), alcohol (n=8), and depression (n=17)] in the regression analysis were excluded to ensure comparability among models **n=1,439**

Participants with nonpositive weights (n=29) were excluded **n=1,410**

**Supplemental Table 1: Cognitive Domain Construction**

| **Cognitive Domain** | **Cognitive tests** |
| --- | --- |
| Memory | Sum of 10-word recall (delayed), 3-word recall (delayed), logical memory II (delayed), story recall - brave man (delayed), 10-word recall (recognition), logical memory (recognition), and constructional praxis (delayed) |
| Executive function | Sum of standard progressive matrices, number series, trial making part B, SDMT, trial making part A, backwards spelling, backwards counting, and letter cancellation |
| Language | Sum of animal naming, object naming TICS, object naming MMSE, 0-2, write a sentence, read and follow command, and object naming CSI-D |
| Visuospatial | Constructional praxis (immediate) task, adjusted to a T-score metric |
| Orientation | Sum of orientation to time and place |

**Standardized the five CD to a mean of 50 and a standard deviation of 10*

**Supplemental Table 2: Associations between food insecurity and cognitive domains stratified by sex**

|  | **β (95% CI)^a*^** | ***P-interaction*** |
| --- | --- | --- |
| **Memory** |  | ***0.0366*** |
| **Female** | 0.76 (-1.30, 2.82) |  |
| **Male** | -1.79 (-4.35, 0.77) |  |
| **Executive Functioning** |  | ***0.0464*** |
| **Female** | -1.05 (-2.21, 0.11) |  |
| **Male** | **-2.14 (-3.95, -0.33)** |  |
| **Language** |  | *0.4086* |
| **Female** | 0.45 (-0.72, 1.62) |  |
| **Male** | -0.13 (-3.02, 2.76) |  |
| **Visuospatial** |  | *0.0657* |
| **Female** | 0.76 (-2.63, 4.15) |  |
| **Male** | -0.28 (-3.98, 3.43) |  |
| **Orientation** |  | *0.6656* |
| **Female** | -0.65 (-3.79, 2.49) |  |
| **Male** | -0.03 (-3.29, 3.22) |  |

***All covariates were drawn from the 2012 core HRS or 2013 HCNS surveys.**

**^a^Adjusted for age, race/ethnicity, total net worth, education, marital status, physical activity, BMI, smoking status, CVD, alcohol consumption, and depression status with Bonferroni correction.**

**Supplemental Table 3: Associations between food insecurity and cognitive domains stratified by race and ethnicity**

|  | **β (95% CI)^a*^** | ***P-interaction*** |
| --- | --- | --- |
| **Memory** |  | *0.4311* |
| **White** | -0.74 (-3.03, 1.55) |  |
| **Black** | 0.87 (-3.65, 5.38) |  |
| **Hispanic** | -0.70 (-2.66, 1.25) |  |
| **Other** | 1.98 (-20.21, 24.17) |  |
| **Executive Functioning** |  | *0.1609* |
| **White** | -1.36 (-3.02, 0.30) |  |
| **Black** | -0.52 (-3.41, 2.36) |  |
| **Hispanic** | **-2.62 (-4.98, -0.26)** |  |
| **Other** | -0.98 (-12.08, 10.12) |  |
| **Language** |  | *0.3062* |
| **White** | 0.44 (-1.15, 2.04) |  |
| **Black** | -0.39 (-7.30, 6.52) |  |
| **Hispanic** | -0.94 (-4.08, 2.19) |  |
| **Other** | 1.56 (-18.52, 21.64) |  |
| **Visuospatial** |  | *0.1560* |
| **White** | 1.92 (-1.31, 5.15) |  |
| **Black** | -2.57 (-9.13, 3.99) |  |
| **Hispanic** | -6.67 (-14.06, 0.71) |  |
| **Other** | 2.07 (-27.32, 31.46) |  |
| **Orientation** |  | *0.2316* |
| **White** | -0.08 (-2.97, 2.81) |  |
| **Black** | 0.24 (-5.80, 6.28) |  |
| **Hispanic** | -1.61 (-5.22, 2.00) |  |
| **Other** | 4.52 (-54.39, 63.42) |  |

***All covariates were drawn from the 2012 core HRS or 2013 HCNS surveys.**

**^a^Adjusted for age, sex, total net worth, education, marital status, physical activity, BMI, smoking status, CVD, alcohol consumption, and depression status with Bonferroni correction.**

**Supplemental Table 4: Associations between food insecurity (including marginal food security) and cognitive domains (n=1,410)**

|  | **Food secure** | **Food insecure** | **Model 1^a**^** | **Model 2^b**^** | **Model 3^c**^** |
| --- | --- | --- | --- | --- | --- |
|  | **Mean (SE)** | **Mean (SE)** | **β (95% CI)** | |  |
| **Memory*** | 51.50 (0.23) | 49.62 (0.42)**^d^** | **-2.45 (-3.69, -1.189)** | -0.41 (-1.81, 0.99) | -0.19 (-1.58, 1.19) |
| **Executive Functioning*** | 51.21 (0.12) | 48.56 (0.34)**^d^** | **-2.99 (-3.98, -2.01)** | **-1.24 (-2.34, -0.14)** | -0.92 (-1.93, 0.10) |
| **Language*** | 50.48 (0.16) | 49.53 (0.42) | -1.12 (-2.40, 0.16) | 0.18 (-1.04, 1.40) | 0.55 (-0.65, 1.75) |
| **Visuospatial*** | 51.98 (0.34) | 49.11 (1.11)**^d^** | **-3.25 (-6.29, -0.22)** | -0.31 (-3.17, 2.55) | 0.38 (-2.34, 3.10) |
| **Orientation*** | 50.74 (0.27) | 50.10 (0.49) | -0.99 (-2.38, 0.41) | 0.29 (-1.21, 1.79) | 0.48 (-1.30, 2.27) |

***Standardized to mean 50 and SD of 10.**

****All covariates were drawn from the 2012 core HRS or 2013 HCNS surveys.**

**^a^Adjusted for age and sex with Bonferroni correction.**

**^b^Adjusted for age, sex, race/ethnicity, total net worth, education, and marital status with Bonferroni correction.**

**^c^Adjusted for age, sex, race/ethnicity, total net worth, education, marital status, physical activity, BMI, smoking status, CVD, alcohol consumption, and depression status with Bonferroni correction.**

**^d^Means significantly different at P<0.05**

**Supplemental Table 5: Associations between food insecurity and cognitive domains adjusting for childhood SES (n=1,308)**

|  | **Food secure** | **Food insecure** | **Model 1^a**^** | **Model 2^b**^** | **Model 3^c**^** |
| --- | --- | --- | --- | --- | --- |
|  | **Mean (SE)** | **Mean (SE)** | **β (95% CI)** | |  |
| **Memory*** | 51.40 (0.23) | 49.03 (0.64)**^d^** | **-2.71 (-4.54, -0.87)** | -0.68 (-2.81, 1.46) | -0.36 (-2.38, 1.66) |
| **Executive Functioning*** | 51.15 (0.14) | 47.53 (0.41)**^d^** | **-3.82 (-5.09, -2.55)** | **-2.14 (-3.62, -0.67)** | **-1.58 (-2.98, -0.18)** |
| **Language*** | 50.54 (0.15) | 49.20 (0.33)**^d^** | -1.46 (-3.08, 0.16) | -0.03 (-1.43, 1.36) | 0.43 (-0.87, 1.73) |
| **Visuospatial*** | 51.94 (0.33) | 48.62 (1.05)**^d^** | **-3.57 (-6.50, -0.64)** | -0.53 (-3.38, 2.32) | 0.58 (-2.09, 3.25) |
| **Orientation*** | 50.71 (0.27) | 48.93 (0.87) | -1.99 (-4.27, 0.29) | -0.78 (-2.91, 1.36) | -0.42 (-2.81, 1.97) |

***Standardized to mean 50 and SD of 10.**

****All covariates were drawn from the 2012 core HRS or 2013 HCNS surveys.**

**^a^Adjusted for age and sex with Bonferroni correction.**

**^b^Adjusted for age, sex, race/ethnicity, total net worth, education, and marital status with Bonferroni correction.**

**^c^Adjusted for age, sex, race/ethnicity, total net worth, education, marital status, physical activity, BMI, smoking status, CVD, alcohol consumption, depression status, and childhood SES with Bonferroni correction.**

**^d^Means significantly different at P<0.05**

**Supplemental Table 6: Associations between risk/protective factors and executive functioning (n=1,410)**

|  | **Model 1^a**^** | **Model 2^b**^** | **Model 3^c**^** |
| --- | --- | --- | --- |
|  | **β (95% CI)** | | |
| **Food Insecurity** | **-3.50 (-4.62, -2.37)** | **-1.87 (-3.16, -0.58)** | **-1.47 (-2.65, -0.28)** |
| **Age** | **-0.20 (-0.26, -0.15)** | **-0.19 (-0.23, -0.14)** | **-0.19 (-0.24, -0.14)** |
| **Education** |  |  |  |
| **Less than high school** | N/A | reference | reference |
| **High school graduate** | N/A | **2.91 (1.81, 4.02)** | **2.91 (1.84, 3.98)** |
| **Some college/college graduate** | N/A | **3.91 (2.82, 4.99)** | **3.81 (2.63, 4.98)** |
| **Post-college** | N/A | **4.78 (3.31, 6.24)** | **4.54 (3.02, 6.07)** |

***Standardized to mean 50 and SD of 10.**

****All covariates were drawn from the 2012 core HRS or 2013 HCNS surveys.**

**^a^Adjusted for age and sex with Bonferroni correction.**

**^b^Adjusted for age, sex, race/ethnicity, total net worth, education, and marital status with Bonferroni correction.**

**^c^Adjusted for age, sex, race/ethnicity, total net worth, education, marital status, physical activity, BMI, smoking status, CVD, alcohol consumption, and depression status with Bonferroni correction.**

**^d^Means significantly different at P<0.05**
